# Chronic metabolic stress impairs lymphatic contractility via activation of KATP channels in a mouse model of Type-2 diabetes

**DOI:** 10.3389/fphys.2025.1558763

**Published:** 2025-04-30

**Authors:** Jorge A. Castorena-Gonzalez, Hae Jin Kim, Michael J. Davis

**Affiliations:** ^1^ Department of Pharmacology, School of Medicine, Tulane University, New Orleans, LA, United States; ^2^ Department of Medical Pharmacology and Physiology, School of Medicine, University of Missouri, Columbia, MO, United States

**Keywords:** lymphatic vessel, contractile dysfunction, db/db, lymphedema, KATP

## Abstract

**Introduction:**

Chronic metabolic stress is a common underlying factor of multiple diseases, including obesity, type II diabetes, and metabolic syndrome. Lymphatic dysfunction, including valve defects, impaired contractile activity, and hyperpermeability, is also associated with these same diseases. We recently reported that acute metabolic stress leads to activation of KATP channels in lymphatic muscle cells, resulting in impairment of the intrinsic lymphatic pacemaker that drives their spontaneous contractions and active lymphatic pumping.

**Methods:**

In the present study, we tested whether lymphatic contractile dysfunction occurs in the db/db mouse, a model of metabolic syndrome, and, if so, to what extent dysfunction might be mediated by KATP channel activation. Contractile function was assessed *ex vivo* in cannulated and pressurized popliteal collecting lymphatics from age-matched db/db mice or their BKS controls (from males and females at 18–20 weeks of age).

**Results:**

Vessels from db/db mice exhibited pressure-dependent spontaneous contractions that were significantly reduced in amplitude, frequency, and calculated fractional pump flow at all tested pressures in the range 0.5 to 5 cmH_2_O, compared to BKS controls. The impaired contractile function of lymphatic vessels from db/db mice was improved by the KATP channel inhibitor glibenclamide (GLIB) at a concentration (1 mM) previously shown to have little or no off-target effects on lymphatic function. Because db/db mice are both obese and have elevated blood glucose levels, we tested whether elevated glucose per se altered contractile function. In glucose levels characteristic of diabetic animals (23 mM), the contraction frequency and fractional pump flow of lymphatic vessels from WT mice were significantly decreased compared to those observed in normal (5 mM) glucose concentrations. The equivalent concentration of mannitol, an osmotic control, did not result in any significant changes in lymphatic contractile function. Lymphatic dysfunction induced by high glucose was rescued by GLIB (1 mM), and lymphatic vessels from Kir6.1^−/−^ mice were largely resistant to the inhibitory effects of high glucose.

**Discussion:**

Our results suggest that a substantial fraction of lymphatic contractile impairment in db/db mice is mediated by the activation of KATP channels in lymphatic muscle cells, in part due to chronic metabolic stress associated with elevated glucose.

## 1 Introduction

The lymphatic system plays fundamental roles in the reabsorption and transport of excess interstitial fluid back into the central veins, the coordination of efficient and effective immune responses, the absorption and transport of lipids, as well as tissue-specific roles that contribute to the normal functioning of tissues and organs (Liao and Padera, 2013; Randolph et al., 2017; Petrova and Koh, 2018; 2020; Stritt et al., 2021; Angeli and Lim, 2023; Davis et al., 2024). It is now recognized that dysfunction of the lymphatic system is not only associated with various diseases, but it can also be a key determining factor in their development and progression. Relevant to the central topic of this study, obesity, metabolic syndrome, and type-2 diabetes have been bidirectionally associated with secondary lymphedema (Greene et al., 2012; Mehrara and Greene, 2014; Greene, 2016; Ashing et al., 2020; Figenshau and Lindquist, 2020; Kataru et al., 2020; de Sire et al., 2021; Rockson, 2021; Sudduth and Greene, 2022b; a).

Secondary lymphedema is more prevalent in breast-cancer patients following surgical (i.e., lymph node dissection), chemo- and radio-therapeutic intervention (Rockson, 2023; Siotos et al., 2024); and obesity, type-2 diabetes, and metabolic syndrome are established risk factors (Helyer et al., 2010; Tsai et al., 2018; Duyur Cakit et al., 2019; Rockson, 2020; Siotos et al., 2024). Studies conducted using various animal models of obesity, metabolic syndrome, and type-2 diabetes have provided important insights into some of the mechanisms leading to lymphatic dysfunction in association with these diseases. For instance, we recently demonstrated that lymphatic valve deficiency results from diet-induced obesity in WT mice; and diet-induced obese ApoE-KO mice display an even more severe dysfunctional phenotype including hyperpermeability and contractile impairment, in addition to profound valve dysfunction (Castorena-Gonzalez, 2022; Davis et al., 2022b). Previously, *in vivo* studies on diet-induced obese WT mice uncovered a reduced contractility of collecting lymphatics (i.e., decreased frequency) and impaired response to mechanostimulation (Blum et al., 2014), as well as impaired lymphatic fluid transport and impaired dendritic cell migration (Weitman et al., 2013). Increased lymphatic vascular permeability was demonstrated in obese and type-2 diabetic mice (Scallan et al., 2015). In a rat model of obesity and metabolic syndrome, studies have demonstrated an association with various aspects of dysfunction in collecting lymphatic vessels, including impairment of their intrinsic contractility, inward remodeling, and blunted flow-mediated responses. These animals were also characterized by increased expression of inflammatory genes (i.e., TNFα, SREBP1C), decreased expression of nitric oxide synthase (eNOS), decreased expression of the sarcoplasmic reticulum Ca^2+^ ATPase pump (i.e., SERCA2a), and increased accumulation of CD163+ MHCII + macrophages (Chakraborty et al., 2010; Zawieja et al., 2012; Zawieja et al., 2016a; Zawieja et al., 2016b; Lee et al., 2020; Lee et al., 2022).

Although such studies have provided important insights into some of the underlying mechanisms of lymphatic dysfunction in obesity, metabolic syndrome, and type-2 diabetes, much remains to be fully elucidated. The significance of exploring novel mechanisms of lymphatic system dysfunction in these metabolic diseases is highlighted by the increasing number of patients afflicted with secondary lymphedema, in association with the global obesity epidemic, and the lack of pharmacological therapies to reverse or ameliorate lymphatic dysfunction.

In a recent study, we demonstrated that acute metabolic stress leads to lymphatic dysfunction mediated by reduced mitochondrial ATP production and increased ROS production, which inhibit the ionic pacemaking mechanisms that control the spontaneous contractility of lymphatic muscle cells (Kim et al., 2024). Interestingly, gain of function mutations in ATP-sensitive potassium channels (i.e., KATP channels) are known to cause Cantú syndrome and a consistent phenotype of Cantú patients is the development of primary lymphedema resulting from contractile impairment of collecting lymphatic vessels (Davis et al., 2020; Davis et al., 2022a; Davis et al., 2023). Altogether, these findings prompted us to test the hypothesis that in metabolic diseases, including obesity, metabolic syndrome, and type-2 diabetes, chronic metabolic stress results in activation of KATP channels leading to lymphatic dysfunction.

Therefore, in this study, we utilized db/db mice, a well-known and established model of type-2 diabetes, obesity, and metabolic syndrome, to determine if contractile dysregulation is present, and if so, determine the extent to which activation of KATP channels contributes. Furthermore, since high blood glucose levels are an important characteristic of these mice, we assessed high glucose as a potential significant mediator of the underlying metabolic stress in this process.

## 2 Materials and methods

### 2.1 Animals

WT (C57BL/6J, Cat.No. 000664), db/db (BKS.Cg-Dock7^m^+/+Lepr^db^/J, Cat.No. 000642), and BKS (C57BLKS/J, Cat.No. 000662) male and female mice were purchased at 6 weeks of age from the Jackson Laboratory–Bar Harbor, MA, USA (JAX). Kir6.1^−/−^ mice were a gift from Susumu Seino (Kobe University, Kobe, Japan). Mice were housed at a temperature of 22–25^o^C under a 12-h light/dark cycle with uninterrupted access to food (regular chow) and water until terminal tissue collection and *ex vivo* experimentation were performed. In preparation for terminal dissection, mice were anesthetized by means of intraperitoneal injection of Ketamine/Xylazine (100/10 mg/kg) and placed on a heated pad for blood collection and tissue dissection. Adequate levels of anesthesia were ensured by continuous monitoring for indicators of pain and by assessing loss of pedal and pinna reflexes. Following dissection, animals were euthanized via overdose of Ketamine/Xylazine and subsequent cervical dislocation.

Age-matched db/db and BKS mice were utilized at 18–20 weeks of age. At this age, db/db mice display continued hyperinsulinemia, in addition to sustained hyperglycemia. Kir6.1^−/−^ showed a high mortality rate beyond 3–4 months of age, and therefore, experiments on Kir6.1^−/−^ and their corresponding WT controls were performed at 6–10 weeks of age. Experiments were conducted in male and female mice in equal ratios. Initially, the data were analyzed separately; however, once we determined that the contractile function was similar in both sexes for the different animal groups, the data were combined.

### 2.2 Assessment of blood glucose levels

With a mouse under anesthesia, unfasted blood glucose concentration was assessed from a droplet of blood collected after a small incision of the tail dorsal caudal vein. Glucose concentration was determined using a glucose meter.

### 2.3 Vessel isolation, pressure myography, and data acquisition

Popliteal afferent collecting lymphatic vessels were isolated as previously described (Scallan and Davis, 2013; Scallan et al., 2015; Castorena-Gonzalez et al., 2018; Zawieja et al., 2018). Briefly, starting with the mouse in the prone position laying on a heated tissue dissection/isolation pad, the superficial saphenous vein was exposed by making a proximal-to-distal incision along the calf area beginning at the ankle and the popliteal afferent lymphatic vessels that run along this major vein were isolated. Then, each popliteal lymphatic tissue was pinned down onto the Sylgard-coated surface of a dissection chamber filled with Krebs-BSA where most of the adipose and connective tissues surrounding the vessel were micro-dissected. Subsequently, a partially clean vessel segment was transferred to an observation chamber filled with Krebs-BSA solution. The vessel was cannulated and pressurized to three cmH_2_O, under no-flow conditions, using two glass micropipettes (inside diameter was ∼40–50 µm). To ensure accurate diameter tracking, the cannulated vessel segment was further cleared of remaining connective and adipose tissue. The observation chamber with cannulated lymphatic vessel segment was transferred onto the XY-stage of an inverted microscope for experimentation. Polyethylene tubing (PE-190) was attached to the back of each glass micropipette and then connected to a microfluidic flow control system (Elveflow OB1 MK3, Paris) with attached low-pressure transducers. Consistent with previous studies (Davis et al., 2011; Lapinski et al., 2017; Castorena-Gonzalez et al., 2018; Castorena-Gonzalez et al., 2020), to minimize longitudinal bowing and associated diameter-tracking artifacts at higher intraluminal pressures, input (P_input_) and output (P_output_) pressures were briefly set to the maximum pressure to be included in the experimental protocol, and the vessel segment was stretched axially to remove longitudinal slack. Before starting an experimental protocol, the vessel was then allowed to equilibrate at 37°C for 30–60 min at P_input_ = P_output_ = 3 cmH_2_O while constantly superfused with Krebs buffer (without BSA) using a peristaltic pump at a rate of 0.5 mL/min. Lymphatic vessels usually began displaying spontaneous contractions within 15–30 min during warm up. Custom-written LabVIEW programs (National Instruments; Austin, TX) were employed for pressure control, as well as real-time vessel diameter tracking and acquisition of video (Davis, 2005). Videos were recorded in brightfield mode at 30 fps using a Basler A641fm camera.

### 2.4 Solutions and chemicals

During microdissection and cannulation of lymphatic segments, a Krebs-BSA buffer was utilized. It contained: 146.9 mM NaCl, 4.7 mM KCl, 2 mM CaCl_2_·2H_2_O, 1.2 mM MgSO_4_, 1.2 mM NaH_2_PO_4_·H_2_O, 3 mM NaHCO_3_, 1.5 mM Na-HEPES, 5 mM D (+)-glucose, and 0.5% BSA (pH = 7.4). A similar Krebs buffer without BSA was utilized for constant superfusion of the lymphatic vessels during warm up and experimental protocols. To determine the maximum passive diameter of a vessel as a function of intraluminal pressure, a Ca^2+^-free Krebs buffer (where 3 mM EGTA replaced calcium) was used. All reagents in the different solutions/buffers, as well as D (+)-glucose (Cat.No.: G5767), and D-mannitol (Cat.No.: M4125) were purchased from Sigma-Aldrich (St. Louis, MO). Glibenclamide (Cat.No.: 0911) was purchased from Tocris Bioscience.

### 2.5 Assessment of lymphatic contractile function

The contractile activity of lymphatic vessels was assessed as a function of intraluminal pressure (i.e., P_input_ = P_output_ = 3, 2, 1, 0.5, 3, and 5 cmH_2_O). For each vessel, the functional response to changes in intraluminal pressure was determined under control conditions and subsequently in the presence of glibenclamide (GLIB, 1 µM), a concentration previously shown to have little or no off-target effects (Kim et al., 2021). The functional responses to different concentrations of glucose and mannitol were assessed in lymphatic vessels from WT animals under no-flow conditions at three cmH_2_O. Following each experimental protocol, the maximum passive diameter of a given vessel at the different tested levels of intraluminal pressure was determined after superfusing the vessel with a Ca^2+^-free Krebs buffer for 30 min. Following experimentation, custom-written analysis programs were used to detect peak end-diastolic diameter (EDD), end-systolic diameter (ESD), contraction amplitude (AMP), and contraction frequency (FREQ) on a contraction-by-contraction basis. In cases when FREQ was zero, no value of AMP was recorded. These parameters were average over a 5-min period and then used to calculate other parameters commonly employed to characterize the contractile function of lymphatic vessels as follows:

Contraction Amplitude=EDD−ESD

$$\mathrm{Normalized}\;\mathrm{Contraction}\;\mathrm{Amplitude}=\left(\frac{EDD-ESD}{D_{\mathrm{Max}}}\right)\times \;100\%$$

$$Ejection\;Fraction\;\left(EF\right)=\frac{{EDD}^{2}-{ESD}^{2}}{{EDD}^{2}}$$

$$Fractional\;Pump\;Flow\;\left(FPF\right)=FREQ\;\times \;EF$$

### 2.6 Statistical analyses

The number N refers to the number of animals per group that were included in this study, while *n* refers to the total number of experiments (i.e., lymphatic segments tested independently). In most cases, more than one lymphatic segment from the same mouse was successfully studied. A description of the specific statistical tests implemented for each data set is included in each figure caption. Normality of the data distributions was assessed using the Shapiro-Wilk and Kolmogorov-Smirnov tests. Results are reported as mean ± SEM with significance set at p < 0.05. All statistical analyzes were performed using GraphPad Prism 10.4.1.

## 3 Results

### 3.1 Activation of KATP channels contributes to the contractile impairment of collecting lymphatic vessels in db/db mice

**FIGURE 1:**
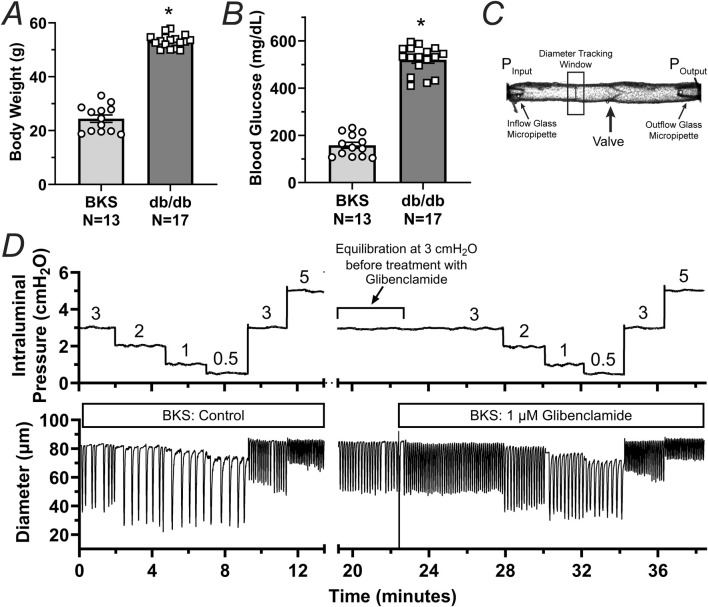
Phenotypic characterization and experimental protocol for the assessment of the contractile function of lymphatic vessels from BKS (control) and db/db mice. **(A, B)** Body weight and unfasted blood glucose levels in db/db (N = 17) and BKS (N = 13) mice respectively. **(C)** Diagram depicting a single-valve isolated, cannulated, and pressurized popliteal afferent collecting lymphatic vessel from a BKS mouse. This panel highlights the inflow and outflow glass micropipettes and their corresponding pressures. **(D)** Representative trace of internal diameter displaying the contractile activity of a lymphatic vessel from a BKS mouse under control conditions and in the presence of glibenclamide (GLIB, 1 μM). Data in panels A and B are presented as mean ± SEM. Statistical differences were determined using parametric t tests with significance defined at *p < 0.05.

**FIGURE 2:**
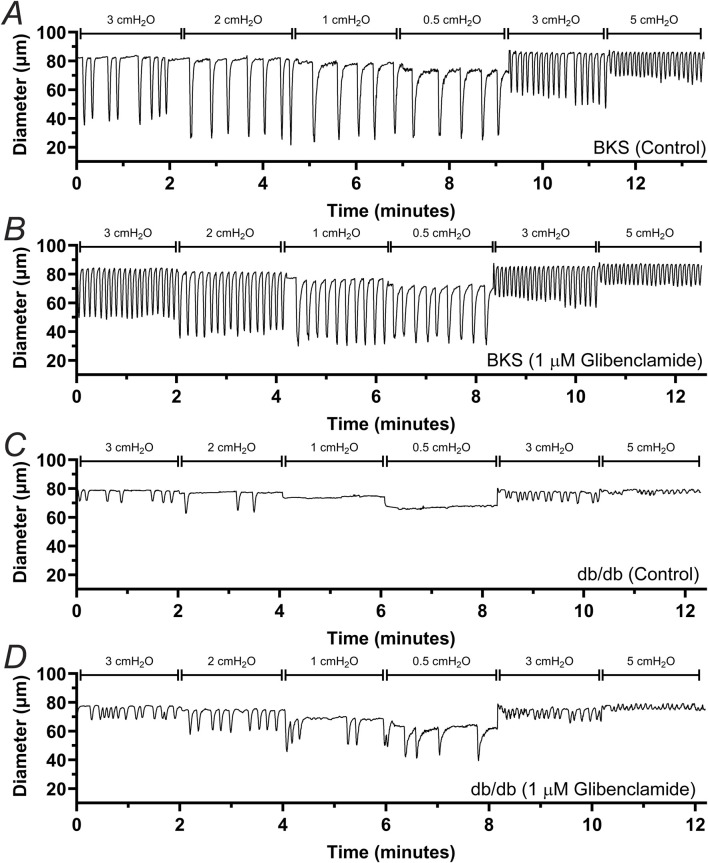
Effect of KATP channel inhibition by GLIB on the contractile activity of lymphatic vessels from BKS and db/db mice. Representative traces depicting the contractile response to different levels of intraluminal pressure for lymphatic vessels from BKS **(A, B)** and db/db mice **(C, D)**. Functional response was assessed under control conditions **(A, C)** and in the presence of GLIB **(B, D)**.

**FIGURE 3:**
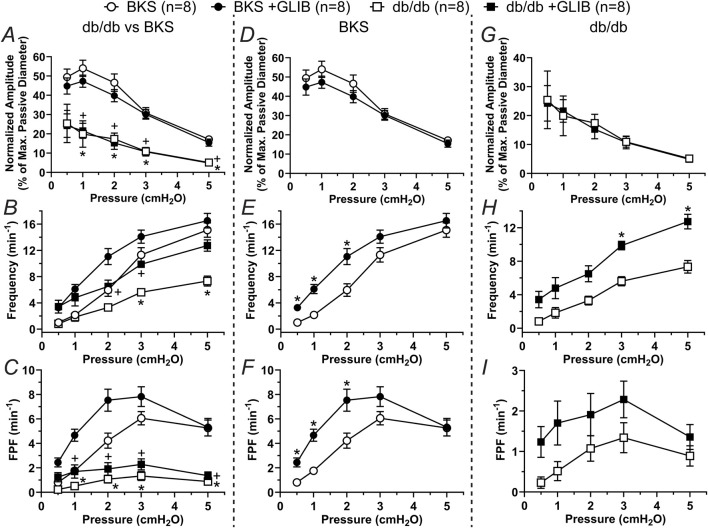
KATP channel inhibition by GLIB enhances the contractility of lymphatic vessels from BKS mice and partially rescues the lymphatic contractile impairment of lymphatic vessels from db/db mice. Summary data of contractile parameters as a function of intraluminal pressure. **(A–C)** Normalized amplitude, frequency, and FPF comparing all groups. In these panels, the symbols indicate significant difference between db/db and BKS in control conditions (*) and in the presence of GLIB (+). **(D–F)** Contractile parameters for lymphatic vessels from BKS mice in control conditions and with GLIB. **(G–I)** Contractile parameters for lymphatic vessels from db/db mice in control conditions and with GLIB. Data are presented as mean ± SEM. Statistical significance was determined at p < 0.05 using two-way ANOVA followed by Tukey *post hoc* test for multiple comparisons for data in panels **(A–C)** or Šídák for data in panels **(D, I)**.

To determine whether db/db mice display lymphatic contractile deficiencies, we characterized the contractile function of isolated, cannulated, and pressurized popliteal afferent collecting lymphatic vessels from db/db mice (N = 17) and BKS controls (N = 13). Consistent with many other studies, db/db mice displayed a significantly increased body weight (53.2 ± 0.6 g) versus that of BKS mice (24.4 ± 1.4 g, Figure 1A), and displayed significantly higher blood glucose levels, i.e., 519.9 ± 14.25 mg/dL and 157.7 ± 13.0 mg/dL, respectively (Figure 1B). The contractile activity of lymphatic vessels from these mice was assessed in the absence of intraluminal flow, and as a function of intraluminal pressure (i.e., P_Input_ = P_Output_ = 3, 2, 1, 0.5, 3, and 5 cmH_2_O, Figure 1C) in control conditions, as well as in the presence of the K_ATP_ channel inhibitor Glibenclamide (GLIB, 1 µM). A representative example of a vessel from a BKS animal is shown in Figure 1D. At the end of each experimental protocol, the maximum passive diameter of each vessel was determined in a Ca^2+^-free Krebs buffer at the different levels of intraluminal pressure. Although not statistically significant, lymphatic vessels from db/db mice were 7.6% ± 0.7% smaller in diameter than those in the BKS group. Consistent with our previously published data using popliteal lymphatic vessels from WT mice, lymphatic vessels from BKS mice displayed rhythmic and robust contractions and high sensitivity to changes in intraluminal pressure, with lower frequency and larger amplitude contractions at low levels of pressure, and higher frequency and lower amplitude contractions as pressure increased (Figure 2A). In contrast, lymphatic vessels from db/db mice displayed significantly impaired contractility as evident by relatively low amplitude, low frequency, erratic contractions (Figure 2C). Inhibition of KATP channels with GLIB (1 µM) further induced significant increases in the contractile frequency and calculated fractional pump flow (FPF) in lymphatic vessels from BKS mice (Figures 2B, 3A–F); and importantly, GLIB partially restored lymphatic contractions of vessels from db/db mice (Figures 2D, 3A–C,G–I), as indicated by the significantly increased frequency and partially enhanced fractional pump flow. GLIB did not induce any significant changes in mean normalized contraction amplitude in either genotype.

**FIGURE 4:**
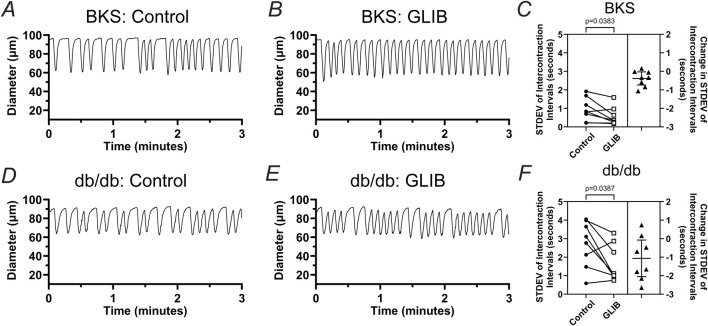
Inhibition of KATP channels by GLIB increases the rhythmicity of lymphatic contractions in vessels from BKS and db/db mice. Representative traces of the contractile activity (at 3 cmH_2_O of intraluminal pressure) of lymphatic vessels from **(A, B)** BKS or **(D, E)** db/db mice under control conditions, and upon treatment with GLIB. **(C, F)** Standard deviation (STDEV) of the intercontraction intervals in control conditions and in the presence of GLIB for lymphatic vessels from BKS and db/db groups respectively. STDEV is used as a measurement of the variability of the intercontraction intervals, and therefore, an indicator of contractile rhythmicity, i.e., lower STDEV values indicative of higher rhythmicity, while higher STDEV values are indicative of erratic non-rhythmic contractions. Data is presented as paired STDEV values per vessel in control conditions and with GLIB, as well as the change in STDEV. Statistical significance was determined using a paired t-test with significance defined at *p < 0.05.

In addition to an increase in contraction frequency, lymphatic contractions, in both the BKS and db/db groups, appeared more rhythmic. To assess lymphatic contractile rhythmicity, we measured the intercontraction interval (i.e., time between successive contractions) for ∼15–30 contractions and then utilized the calculated Standard Deviation (STDEV) as a measure of intercontraction interval variability. For simplicity, these additional analyses were conducted only at pressure 3 cmH_2_O. Representative traces of the contractile activity of lymphatic vessels from BKS and db/db mice in control and in the presence of GLIB are shown in Figures 4A, B, D, E. In control conditions, the STDEV of the intercontraction intervals of vessels from the BKS group were significantly smaller than that of vessels from db/db mice, which was indicative of the rhythmic contractions observed in vessels from BKS mice, and the erratic lymphatic contractions in db/db mice. Interestingly, treatment with GLIB resulted in a significant increase in lymphatic vessel rhythmicity in both BKS and db/db groups, as indicated by the paired analyses showing consistent decreases in the STDEV of intercontraction intervals (Figures 4C, F).

### 3.2 High concentrations of glucose induce lymphatic contractile impairment independently of osmotic stress

**FIGURE 5:**
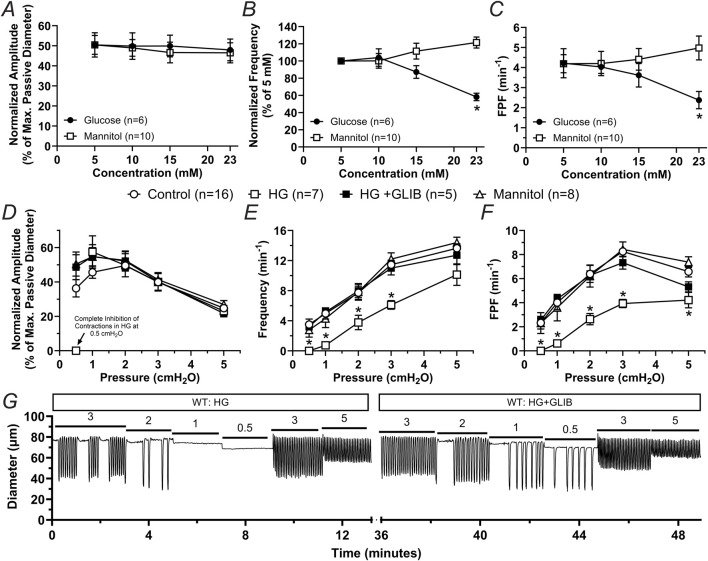
High glucose induces contractile dysfunction mediated by KATP channel activation but independent of osmotic stress in lymphatic vessels from WT mice. **(A–C)** Mean normalized amplitude, normalized frequency, and FPF as a function of increasing concentrations of glucose or mannitol, respectively. Data are presented as mean ± SEM. Statistical significance was determined at p < 0.05 using two-way ANOVA followed by Šídák *post hoc* test for multiple comparisons. **(D–F)** Mean normalized amplitude, frequency, and FPF as a function of intraluminal pressure in control conditions, or in the presence of high glucose (HG, 23 mM), HG + GLIB (1 µM), or mannitol (23 mM). Data are presented as mean ± SEM. Statistical significance was determined at p < 0.05 comparing all groups using two-way ANOVA followed by Tukey *post hoc* test for multiple comparisons. **(G)** Representative trace of the contractile activity of a lymphatic vessel from a WT mouse as a function of intraluminal pressure in HG, as well as in HG and in the presence of GLIB.

In previous studies using db/db mice or diet-induced obese mice, which display obesity and hyperglycemia, we showed that unfasted blood glucose levels reached ∼23 mM (Scallan et al., 2015; Castorena-Gonzalez, 2022). To determine whether higher concentrations of glucose, including concentrations characteristic of diabetic mice, induce contractile dysfunction, we characterized the contractile activity of popliteal lymphatic vessels from WT mice pressurized to 1 cmH_2_O under no-flow conditions after acute exposure to 5 mM (control), 10 mM, 15 mM, and 23 mM of glucose. Glucose concentration did not affect the normalized amplitude of contractions; however, normalized frequency and calculated fractional pump flow (FPF) were significantly decreased (i.e., >40%) in 23 mM glucose (Figures 5A–C). Contractile activity at the lower pressures was particularly affected, consistent with observations made in previous studies (Davis et al., 2020; Davis et al., 2023). To determine if the contractile dysfunction induced by high glucose was merely associated with induced osmotic stress, we tested the functional responses of popliteal lymphatic vessels to equivalent concentrations of the osmotic diuretic, mannitol. The osmotic stress effects of mannitol did not result in any significant changes in the contractile function of these collecting lymphatics, and even led to a slight (22%) increase in contraction frequency (Figures 5A–C).

### 3.3 Lymphatic vessels from KATP deficient animals are largely resistant to contractile dysfunction induced by high glucose

Since inhibition of KATP channels with GLIB partially rescued the contractile dysfunction of lymphatic vessels from db/db mice and significantly increased lymphatic contractile rhythmicity in both BKS and db/db groups (Figures 2–4), we decided to test the extent to which activation of KATP channels plays a role in the lymphatic contractile impairment induced by high glucose (HG, i.e., 23 mM). Consistent with the experiments performed in vessels from db/db mice, the contractile function of lymphatic vessels from WT mice was assessed at different levels of intraluminal pressure under control conditions, HG (23 mM), HG + GLIB (1 µM), or mannitol (23 mM). The contractile function of lymphatic vessels in the presence of mannitol was similar to that of vessels in control conditions. In contrast, HG induced robust contractile impairment (i.e., significantly decreased frequency and FPF). Furthermore, GLIB completely rescued the lymphatic contractile dysfunction induced by HG (Figures 5D–F) even at low pressure levels where HG resulted in complete inhibition of contractions. Representative traces of the contractile activity of a lymphatic vessel from a WT mouse in HG, as well as in HG in the presence of GLIB, are shown in Figure 5G.

**FIGURE 6:**
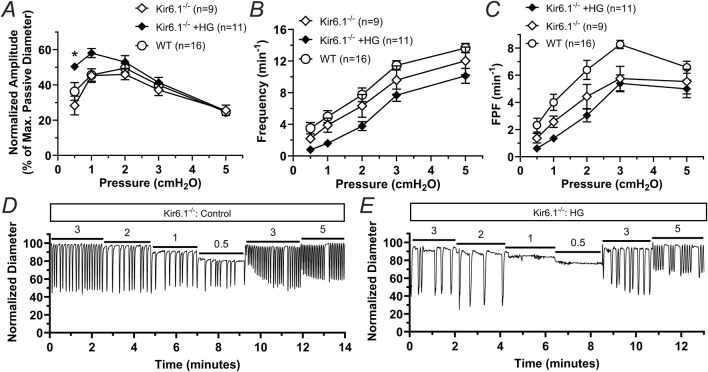
Lymphatic vessels from Kir6.1-deficient mice are largely resistant to lymphatic dysfunction induced by HG. **(A)** Normalized amplitude, **(B)** frequency, and **(C)** FPF as a function of intraluminal pressure for lymphatic vessels from mice lacking Kir6.1 in control conditions or in the presence of high glucose (HG, 23 mM). Data from WT vessels under control conditions, initially presented in Figures 5D–F, is here included again for comparison. Data are presented as mean ± SEM. Statistical significance was determined at p < 0.05 comparing all groups using two-way ANOVA followed by Šídák *post hoc* test for multiple comparisons. Representative traces of the contractile activity as a function of intraluminal pressure of a lymphatic vessel from a Kir6.1^−/−^ mouse under **(D)** control conditions and **(E)** while in the presence of HG.

To further confirm the involvement of KATP channels in the contractile dysfunction induced by HG, we assessed the effects of HG in regulating the contractile function of lymphatic vessels from mice globally deficient in Kir6.1 (i.e., Kir6.1^−/−^ mice lacking KATP channels). The contractile frequency and FPF observed in lymphatic vessels from Kir6.1^−/−^ remained largely unaffected in HG. Interestingly, the normalized contraction amplitude of lymphatic vessels from these KATP deficient mice was significantly increased in the presence of HG in the lower pressures (i.e., 0.5 cmH_2_O and one cmH_2_O), but remained unchanged at higher pressures (i.e., >1 cmH_2_O) (Figures 6A–C). Representative traces of the contractile activity of a lymphatic vessel from a Kir6.1^−/−^ mouse in control conditions and in the presence of HG are shown in Figures 6D, E.

## 4 Discussion

In the present study, we employed db/db mice as a model of type-2 diabetes, obesity, and metabolic syndrome to first determine the extent of lymphatic contractile dysfunction in collecting lymphatic vessels, and then to investigate and elucidate the underlying mechanisms. We found that popliteal lymphatic vessels from db/db mice displayed robust contractile impairment, which could be partially rescued by inhibition of KATP channels by GLIB. In healthy lymphatics from WT mice, metabolic stress induced by HG (similar to those observed in db/db mice) resulted in contractile dysfunction mediated by KATP activation, as it was reversed by GLIB. These observations were finally validated using a global Kir6.1-deficient mouse model. The contractile function of lymphatic vessels lacking KATP channels was highly resistant to the detrimental effects of HG.

Initially, we characterized the contractile function of popliteal afferent collecting lymphatic vessels from db/db mice and their matching BKS controls and demonstrated that profound contractile dysfunction is associated with this model, as evident by an erratic contraction pattern, low amplitude and low frequency contractions. The contractile activity of these vessels was assessed as a function of intraluminal pressure, in a range of pressures spanning the previously determined physiological levels (Czepielewski et al., 2021). While lymphatic vessels from db/db mice still displayed a functional response to increasing pressure, the largely impaired contractions are likely incapable of generating enough propulsive pressure for efficient transport of lymph (evident by the significantly reduced FPF), especially in the presence of any adverse pressure gradient.

Since our previous studies demonstrated that acute metabolic stress is associated with lymphatic dysfunction mediated by activation of KATP channels (Kim et al., 2024), we tested the effects of GLIB and demonstrated that pharmacological inhibition of KATP channels significantly enhanced the otherwise poor contractility of lymphatic vessels from db/db mice. These findings suggest that a component of lymphatic dysfunction in db/db mice is associated with activation of KATP channels.

Furthermore, approximately 37% and 25% of the vessels from db/db mice did not display any contractile activity at the lower range of intraluminal pressures, 0.5 and 1 cmH_2_O respectively; while in the BKS group, only ∼12% of the vessels did not display contractile activity at 0.5 cmH_2_O, but all vessels displayed robust rhythmic contractions at 1 cmH_2_O. Interestingly, inhibition of KATP channels by GLIB induced a significant increase in contractile function in BKS and db/db mice even at these low levels of intraluminal pressure. These observations suggest that the mechanisms underlying lymphatic pacemaking dysfunction in db/db mice not only are dependent on KATP channel activation but also are particularly vulnerable at lower levels of intraluminal pressure.

Results from our previous studies also showed that impaired contractility of LMCs in acute metabolic stress resulted in increased production of reactive oxygen species (ROS) and/or reduced mitochondrial ATP (Kim et al., 2024). Therefore, we hypothesized that a similar mechanism operated in animal models of chronic metabolic stress. The db/db mouse model recapitulates many of the characteristics observed in obesity, type-2 diabetes, and metabolic syndrome, including high blood glucose. We explored the potential role of high glucose as the main driver of lymphatic dysfunction in chronic metabolic stress in db/db mice. In isolated lymphatic vessels from WT mice, high glucose induced significant reduction in contractile frequency, while contraction amplitude remained unchanged, strikingly similar to the effects of acute metabolic stress induced by pharmacological inhibition of the mitochondrial electron transport chain (Kim et al., 2024). In the present study, lymphatic contractile function in lymphatics from WT mice was only significantly impaired at a high glucose level of 23 mM (HG), a level of unfasted glucose displayed in WT mice fed a western diet or in db/db mice (Scallan et al., 2015; Castorena-Gonzalez, 2022). An equivalent concentration of the osmotic diuretic mannitol did not induce any significant changes in lymphatic contractility, suggesting that the lymphatic contractile dysfunction induced by HG is not mediated by osmotic stress. HG completely or nearly-completely inhibited the contractile function of popliteal lymphatic vessels in the pressure range <2 cmH_2_O. This was fully reversed by GLIB, suggesting an important role for KATP channel activation in HG-induced metabolic stress leading to hyperpolarization of the cell membrane of LMCs and subsequent inhibition of the pacemaking activity that regulates the intrinsic lymphatic pumping.

To confirm the direct involvement of KATP channels in acute and chronic metabolic stress induced by HG, as it occurs in db/db mice, we then performed experiments in lymphatic vessels from mice globally deficient in Kir6.1 (i.e., Kir6.1^−/−^). Upon acute stimulation with HG, vessels from these Kir6.1 deficient mice were largely protected against the detrimental effects of HG previously observed in vessels from WT mice. Our previous studies have demonstrated that KATP channel activation occurs only in the LMC layer because 1) mouse LECs do not express functional KATP channels (Davis et al., 2020); and 2) lymphatic vessels from mice with SM-specific inactivation of KATP channels are equally resistant to acute metabolic stress as vessels from Kir6.1^−/−^ mice (Kim et al., 2024). These findings suggest that LMC KATP channels can potentially be targeted therapeutically by GLIB to improve lymphatic contractile function in animal models of chronic metabolic stress, e.g., db/db, diet-induced obesity, and ApoE-KO among others, as well as in mice displaying a gain of function in KATP channels, as happens in Cantú syndrome patients. Future studies are necessary to address these important questions.

## Data Availability

The original contributions presented in the study are included in the article; further inquiries can be directed to the corresponding author.
